# Allele-Specific *KRT1* Expression Is a Complex Trait

**DOI:** 10.1371/journal.pgen.0020093

**Published:** 2006-06-09

**Authors:** Heng Tao, David R Cox, Kelly A Frazer

**Affiliations:** Perlegen Sciences, Mountain View, California, United States of America; Cornell University, United States of America

## Abstract

The differential expression of alleles occurs commonly in humans and is likely an important genetic factor underlying heritable differences in phenotypic traits. Understanding the molecular basis of allelic expression differences is thus an important challenge. Although many genes have been shown to display differential allelic expression, this is the first study to examine in detail the cumulative effects of multiple *cis*-regulatory polymorphisms responsible for allele-specific expression differences. We have used a variety of experimental approaches to identify and characterize *cis*-regulatory polymorphisms responsible for the extreme allele-specific expression differences of keratin-1 *(KRT1)* in human white blood cells. The combined data from our analyses provide strong evidence that the *KRT1* allelic expression differences result from the haplotypic combinations and interactions of five *cis*-regulatory single nucleotide polymorphisms (SNPs) whose alleles differ in their affinity to bind transcription factors and modulate *KRT1* promoter activity. Two of these *cis*-regulatory SNPs bind transcriptional activators with the alleles on the high-expressing *KRT1* haplotype pattern having a higher affinity than the alleles on the low-expressing haplotype pattern. In contrast, the other three *cis*-regulatory SNPs bind transcriptional inhibitors with the alleles on the low-expressing haplotype pattern having a higher affinity than the alleles on the high-expressing haplotype pattern. Our study provides important new insights into the degree of complexity that the *cis*-regulatory sequences responsible for allele-specific transcriptional regulation have. These data suggest that allelic expression differences result from the cumulative contribution of multiple DNA sequence polymorphisms, with each having a small effect, and that allele-specific expression can thus be viewed as a complex trait.

## Synopsis

Despite the fact that all humans share nearly identical DNA sequences, individuals exhibit tremendous variation in heritable traits, such as height, weight, and skin texture. Recent evidence suggests that expression level differences between different copies (alleles) of a gene contribute to these observed differences in heritable traits. Currently, the mechanisms underlying allele-expression level differences are poorly understood. In this report the authors identified and characterized a set of five single nucleotide polymorphisms (SNPs) contributing to extreme expression differences between keratin-1 *(KRT1)* alleles in humans. Each of the five SNPs is found in a different regulatory sequence in the proximity of *KRT1*. The SNPs cause different copies of the five regulatory sequences to differ in their affinities to bind transcription factors controlling *KRT1* expression. The extreme *KRT1* allele-expression level differences result from the cumulative contributions of these five SNPs which are tightly linked and inherited in two common fixed sets, a low- and a high-expressing set. The study provides important new insights into the complexities of the mechanisms underlying allele-expression level differences. These complexities may explain the difficulties researchers frequently encounter when trying to discover the “causative SNP” in an interval identified as associated with an inherited trait in a genetic study.

## Introduction

Allele-specific expression differences can be identified by comparing the relative levels of exonic single nucleotide polymorphism (SNP) alleles within mRNA samples isolated from unrelated individuals [1–7]. Both *cis*- and *trans*-regulatory polymorphisms contribute to differential allelic expression [6,8,9]. *Cis*-regulatory polymorphisms are in close proximity to the gene being regulated and directly affect the transcription initiation, transcription rate, or transcript stability in an allele-specific manner. In contrast, *trans*-regulatory polymorphisms are not in close physical proximity to the gene being regulated and modify either the expression level or activity of a factor that interacts with *cis*-regulatory sequences. Within an individual mRNA sample, alleles are exposed to the same cellular environment, and thus their differential expression must be due to *cis*-regulatory polymorphisms, whether or not *trans*-regulatory polymorphisms also play a role.

Patterns of differential allelic expression in mRNA samples isolated from unrelated individuals can reveal underlying regulatory mechanisms [10]. When allelic expression differences are strongly influenced by *trans*-regulatory polymorphisms, one exonic SNP allele will be expressed at a higher level in some heterozygous individuals while in different individuals the other exonic SNP allele will be expressed at a higher level. In contrast, when allelic expression differences arise primarily from *cis*-regulatory polymorphism in strong linkage disequilibrium with a gene, the same exonic SNP allele will be expressed at a higher level in all heterozygous individuals.

In a large-scale analysis we previously genotyped approximately 2,000 exonic SNP alleles and measured their relative expression levels using oligonucleotide arrays, to identify genes with differential allelic expression [10]. In that study we showed that keratin 1 *(KRT-1),* which belongs to a large family of intermediate filament protein genes and is normally expressed in keratinocytes in the spinous layer of the epidermis [11,12], has extreme allele-specific expression differences in human white blood cells. Unrelated individuals in the study heterozygous for a selected *KRT1* exonic SNP allele all had the same allele expressed at a higher level, suggesting that the differential allelic expression of *KRT1* is predominantly controlled by *cis*-regulatory polymorphism(s) in strong linkage disequilibrium with the gene.

In this study we set out to analyze the *cis*-regulatory polymorphisms responsible for the expression differences of the *KRT1* alleles in detail. We performed a large number of experimental assays to identify and characterize SNP alleles in a previously defined 26-kb *KRT1* haplotype block that have differential regulatory functions. We found five *cis*-regulatory sequences which contain SNP alleles that differ in their affinity to bind transcription factors and modulate *KRT1* promoter activity. This study provides the first detailed molecular analysis of multiple *cis*-regulatory sequences whose combined effects are responsible for differential expression of two alleles. The results suggest that the *cis*-regulation underlying such expression differences can be highly complex.

## Results

### Differential Expression of *KRT1* in Human White Blood Cells

**Figure 1:**
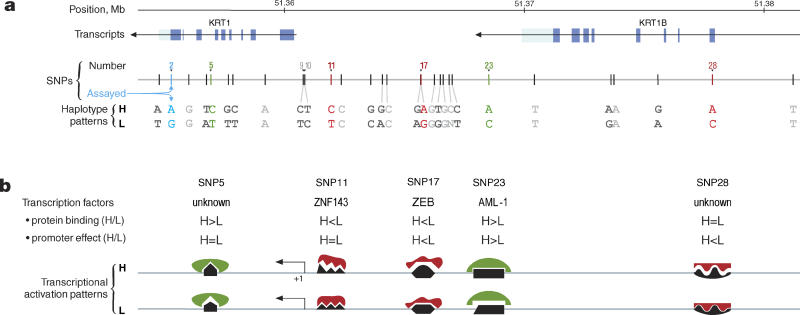
The *KRT1* Haplotype Block (A) The locations of the *KRT1* and *KRT1B* genes and each of the 29 SNPs (represented by a tick mark) are shown, with the SNPs discussed in detail numbered. The SNP alleles of the high- (H) and low- (L) expressing haplotypes are shown, with the 19 SNPs that differ between the two haplotypes highlighted. The exonic SNP (number 2) assayed for differential allelic expression is blue; those shown by transient expression assay to enhance or repress *KRT1* promoter activity are green and red, respectively; and the rest are dark gray. (B) Results from the functional tests for five of the SNPs are summarized. The relative strength of protein binding by the low- and high-expressing SNP alleles is indicated by symbols (>, <, or =). Similarly, the symbols (>, <, or =) indicate the relative degree of increased or decreased *KRT1* promoter activity of the low- and high-expressing SNP constructs. The diagram depicts that *cis*-regulatory SNPs 5 and 23 bind activators (green) as evidenced by increased *KRT1* promoter activity over control constructs, and *cis*-regulatory SNPs 11, 17, and 28 bind repressors (red) as evidenced by decreased *KRT1* promoter activity compared with control constructs. When differential binding or activity is observed, the activators have higher affinity to SNP alleles in the high-expressing haplotype (H), while the repressors have higher affinity to SNP alleles in the low-expressing haplotype (L). The names of proteins with consensus binding sites in the SNP-containing intervals are given.

As *KRT1* has not previously been reported as expressed in white blood cells, we confirmed our original oligonucleotide array results [13] by using real-time PCR to analyze the relative expression levels of the *KRT1* alleles in mRNA extracted from the white blood cells of 36 unrelated individuals. 19 of the samples were heterozygous for the assayed exonic SNP2 in *KRT1* (Figure 1A). Of these, 15 had detectable levels of mRNA and could therefore be used to ascertain relative allelic expression levels (Table 1). In each of the 15 samples the A exonic SNP2 allele was expressed at a higher level than the G exonic allele, and in the majority of samples the expression ratio of A to G was extreme (greater than 8-fold) (Table 1). This consistent expression of the A allele over the G allele suggests that the relative expression levels of the *KRT1* alleles in white blood cells are determined by *cis*-acting factors in strong linkage disequilibrium with the assayed exonic SNP.

**Table 1:**
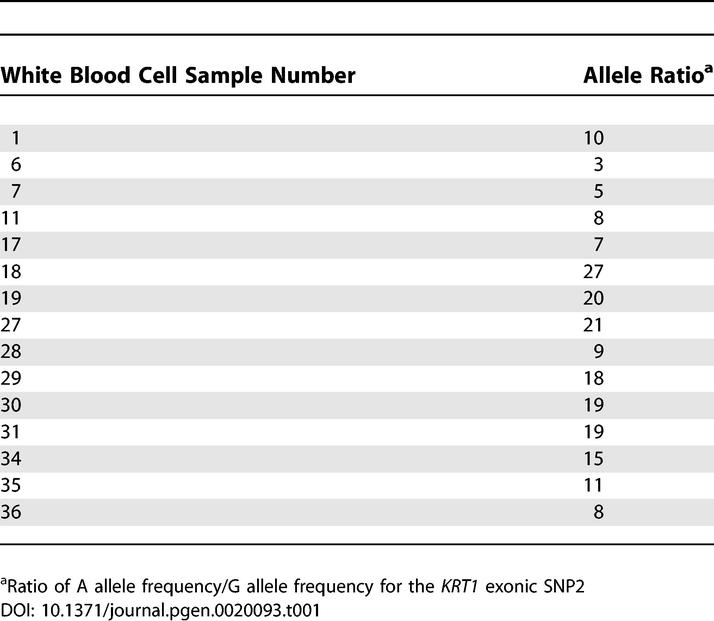
Ratio of *KRT1* Exonic SNP2 Allele Frequencies in Heterozygote White Blood Cell Samples as Determined by Real-Time PCR

### Haplotype Map Construction and Haplotype Patterns in the *KRT1* Block

To identify possible *cis*-regulatory polymorphisms we first identified SNPs in the *KRT1* interval on human Chromosome 12 in linkage disequilibrium with the exonic SNP assayed for differential expression (Figure 1A). To do this we used a comprehensive genome-wide SNP and haplotype map generated by an independent study using ethnically-diverse Coriell samples from the DNA Polymorphism Discovery Resource as previously described [14]. This map showed that *KRT1* is located entirely within a 26-kb haplotype block (Figure 1A), which also contains eight of the nine exons of the *KRT1B* gene. *KRT1B* is a newly identified closely related paralog of human *KRT1* consisting of a similar nine-exon structure that has not yet been functionally characterized [15]. The 29 identified SNPs (Table S1) located within the *KRT1* haplotype block fell into nine haplotype patterns in the Coriell samples (Table S2). One group of four haplotype patterns were minor variants of each other and a separate group of two haplotype patterns were also minor variants of each other. When haplotype patterns with only minor variations were grouped together, the number of observed haplotype patterns in the Coriell samples was reduced to five. 82% of the chromosomes fell into three of the five main haplotype patterns.

### *KRT1* Haplotype Patterns in the 36 White Blood Cell Samples

To determine the relationship between the haplotype patterns and the relative expression levels of the different *KRT1* alleles we determined the haplotype patterns in the 36 white blood cell samples by genotyping seven SNPs (SNPs 2, 5, 6, 11, 17, 23, and 28) that together differentiate the five main *KRT1* haplotypes (Table 2), using real-time PCR. Only four of the five main haplotype patterns present in the Coriell samples were also present in the white blood cell samples. The three most prevalent haplotype patterns observed in the Coriell samples (Table S2) were also the three most prevalent in the 36 white blood cell samples (Table 3). However, the relative frequencies of the haplotypes differ between the two donor populations, suggesting that the ethnic compositions of the two groups differ.

**Table 2:**
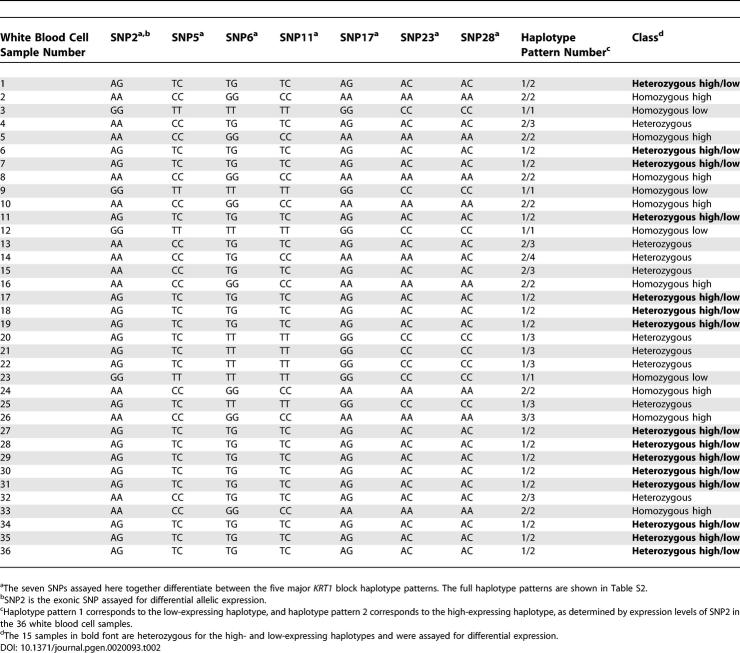
*KRT1* Interval SNP Genotypes in the 36 White Blood Cell Samples

**Table 3:**
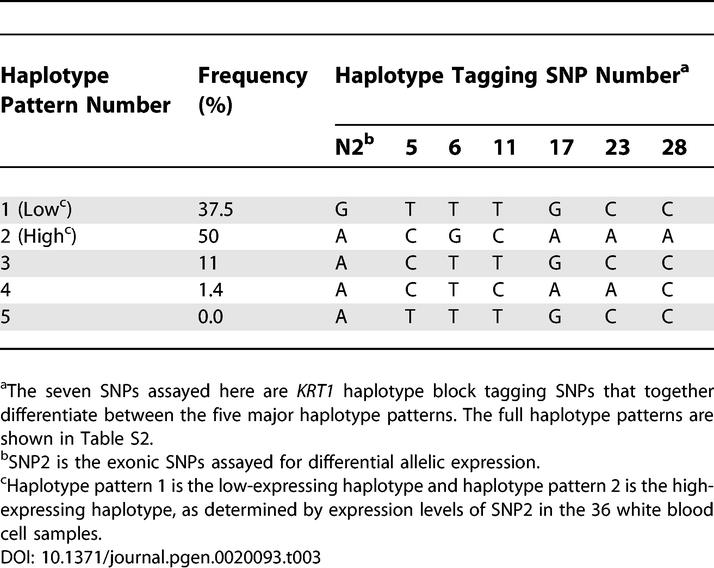
Frequency of *KRT1* Block Haplotype Patterns in the 36 White Blood Cell Samples

The 15 samples that were heterozygous for exonic SNP2 and had detectable levels of *KRT1* mRNA all contained the same two haplotype patterns: pattern number 1, which has the G allele at exonic SNP2, and pattern number 2, which has the A allele at exonic SNP2 (Table 1). In each of these samples, haplotype pattern 2 was expressed at much higher levels than haplotype pattern 1, and thus we defined haplotype pattern 1 as the low-expressing haplotype pattern and haplotype pattern 2 as the high-expressing haplotype pattern. In contrast, the four samples heterozygous for exonic SNP2 that did not express *KRT1* contained haplotype patterns 1 and 3. These data suggest that in human white blood cells *KRT1* haplotype pattern 2 is expressed at significantly higher levels than haplotype patterns 1 or 3. We were unable to examine the relative expression levels of haplotype patterns 4 and 5 due to the fact that in this population the only individual containing pattern 4 was not heterozygous for *KRT1* exonic SNP2 and pattern 5 was not observed. The high number of white blood cell samples heterozygous for haplotype patterns 1 and 2 is expected, given the high frequencies of the two patterns in this population: 37.5% and 50%, respectively.

### Identification of Protein-Binding SNP Intervals

**Figure 2:**
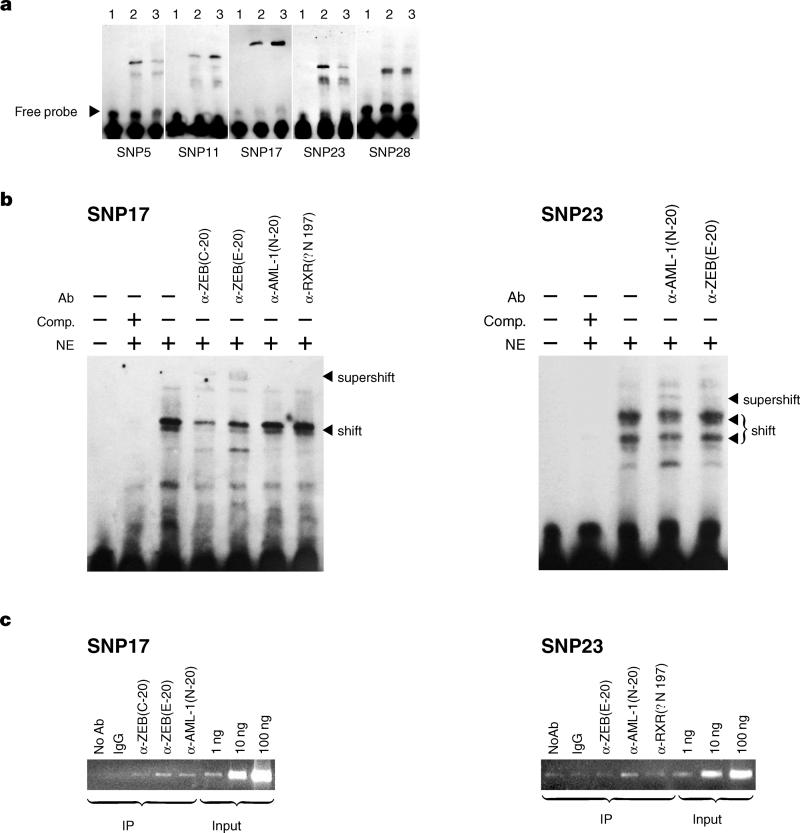
DNA-Protein Binding Studies (A) EMSA analyses for SNPs 5, 11, 17, 23 and 28. Lane 1 shows the high-expressing oligonucleotide incubated without nuclear extract. Lanes 2 and 3 show the high- and low-expressing SNP allele oligonucleotides, respectively, incubated with nuclear extract. (B) Competitor studies and antibody-mediated supershift assays. Biotin-labeled oligonucleotides corresponding to the 25-bp sequences immediately surrounding SNPs 17 and 23 were incubated with (+) or without (−) nuclear extract (NE), 100-fold excess unlabeled oligonucleotide competitor (Comp.), and the specific antibodies (Ab) indicated. (C) PCR assays with primer pairs specific for the SNP17 and SNP23 intervals and DNA that was immunoprecipitated with antibodies against ZEB and AML-1 or mock immunoprecipitated without antibody (No Ab), with anti-rabbit IgG only, or with antibody against retinoid X receptor (RXR). Positive controls consisting of 1 ng, 10 ng, and 100 ng of whole lysate DNA were amplified in parallel.

To identify the *cis*-regulatory polymorphisms responsible for the extreme allele-specific expression differences of the *KRT1* gene, we focused on the SNPs that differentiate the low- and high-expressing haplotype patterns. Of the 29 SNPs in the *KRT1* block interval, 19 differ between the low- and high-expressing haplotype patterns (Figure 1A). We used several experimental techniques to identify which of the 19 SNPs, whose alleles differ between the low- and high-expressing haplotypes, are involved in regulating the differential expression of these haplotypes. First, we performed electrophoretic mobility shift assays (EMSAs) to examine the SNP intervals for effects on DNA-protein interactions. We incubated nuclear extracts from an epithelial cell line with 25-mer double-stranded oligonucleotide probes containing the SNPs in the center positions (Table S3). Under stringent assay conditions only five of the probes, corresponding to SNPs 5, 11, 17, 23, and 28, bound proteins (Figure 2A). Four of these probes showed differential protein binding, with the high-expressing alleles of SNPs 5 and 23 binding more protein than the low-expressing alleles, and the low-expressing alleles of SNPs 11 and 17 binding more protein than the high-expressing alleles. These assays were performed three independent times, with the same relative binding levels between the low- and high-expressing SNP alleles observed.

### TRANSFAC Analysis

To determine if any of the five SNP-containing intervals shown by EMSA to bind proteins contain consensus binding sites, TRANSFAC [16] database searches were performed using the 25-mer oligonucleotide sequences given in Table S3 and the default values of TFSearch (http://www.cbrc.jp/research/db/TFSEARCH.html). SNP intervals 11, 17, and 23 were identified as containing binding sites for Staf (the Xenopus ortholog of the human ZNF143 protein [17]), deltaEF1 (which binds the same sequence, CACCTG, as the human homolog ZEB [18,19]), and AML-1a [20], respectively. These factors all had threshold scores ≥ 90.9 with either the high or low-expressing oligonucleotide sequences and were the only factors whose binding site included the SNP allele. ZNF143 is a transcriptional activator [17,21], while ZEB is a negative regulator of many genes [18,22,23], and AML-1 regulates the expression of genes both negatively and positively [24–26]. Although none of these transcription factors have previously been shown to regulate expression of *KRT1,* ZEB interacts with a co-repressor CtBP [18], which is known to repress the transcription of many epithelial genes [27].

### Specific DNA-Protein Interactions for SNP17 and SNP23 Intervals

The availability of antibodies specific to ZEB and AML-1 allowed us to further examine DNA-protein interactions of the intervals for SNP17 and SNP23. Pre-incubating the epithelial cell line nuclear extract with unlabeled SNP17 or SNP23 competitor oligonucleotides before adding the corresponding biotin-labeled oligonucleotide probes abolished the observed DNA-protein complexes, indicating that they are formed by specific protein-DNA binding interactions (Figure 2B). Simultaneously incubating epithelial cell nuclear extract with the biotin-labeled oligonucleotide and specific antibodies for ZEB-1 produced a supershifted band for SNP17. This supershifted band was observed with the use of two distinct anti-ZEB antibodies, but was not seen when anti-AML or anti-RXR antibodies were used, suggesting that the SNP17 interval binds specifically to ZEB.

Chromatin immunoprecipitation assays showed a clear enrichment of the SNP17 interval with anti-ZEB immunoprecipitates compared with mock immunoprecipitates (Figure 2C), although binding with anti-AML-1 immunoprecipitates is also seen. The fact that protein-DNA interactions between SNP17 interval and AML-1 are not observed in the supershift assay but appear to be present in the immunoprecipitation assay is likely due to that fact that the former uses a DNA interval (25 bp) centered on SNP17 while the later amplifies a 475-bp interval (Table S4) surrounding SNP17. TRANSFAC analysis of this 475-bp sequence shows the region contains a predicted AML-1 binding site 205 bp away from SNP17. Thus, the enrichment of the SNP17 interval with anti-AML-1 immunoprecipitates over mock immunoprecipitates may be due to specific protein-DNA interactions. In aggregate, the TRANSFAC search data, the supershift assay results, and the immunoprecipitate data supports in vivo binding of ZEB to the SNP17 interval.

For the SNP23 interval, simultaneously incubating epithelial cell nuclear extract with the biotin-labeled oligonucleotide and antibodies specific for AML-1 produced a supershifted band (Figure 2B), which is slightly more prominent than the background smear in the same location observed without antibodies and with antibodies specific for ZEB. The chromatin immunoprecipitation assays showed a modest enrichment of the SNP23 interval with anti-AML-1 compared with mock and anti-ZEB immunoprecipitates (Figure 2C). These data provide supporting evidence to the TRANSFAC search data, suggesting that the SNP23 interval binds in vivo with AML-1.

Due to the lack of an appropriate antibody for ZNF143, we were unable to use similar techniques to analyze the SNP11 interval for DNA-protein interactions.

### Functional Characterization of *cis*-Regulatory SNP Intervals

**Figure 3:**
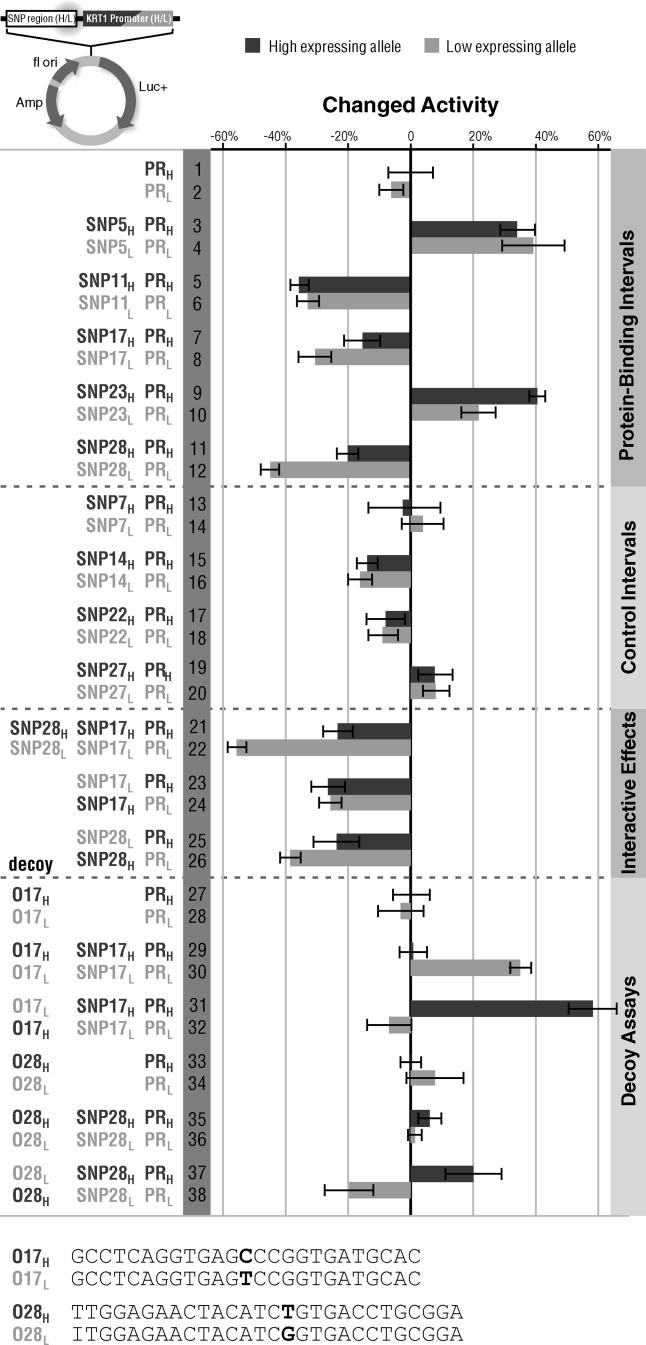
Transient Expression Assays Luciferase reporter constructs containing either the low (L) or the high (H)-expressing *KRT1* promoter (PR_H or L_) or the promoter combined with SNP-containing interval(s) are shown on the left (numbered 1–26). Relative *KRT1* promoter activity of constructs 2–26 is expressed in % versus control construct PR_H_ (assay 1). The relative luciferase activities represent the mean ± standard deviation of two to three independent experiments performed in triplicate or quadruplicate. Double-stranded decoy oligonucleotides, O17_H or L_ and O28_H or L_ (sequences shown at bottom), co-transfected with the *KRT1* promoter alone and in various combinations with the H and L SNP17 and SNP28; constructs are shown for numbers 27–38. The relative *KRT1* promoter activity of constructs 28–32 is expressed in % versus control construct 27, the relative activity for constructs 34–38 is expressed in % versus control construct 33.

To examine how the five SNP-containing intervals shown by EMSA to bind proteins affect in vivo *KRT1* promoter expression, we generated a series of 20 luciferase reporter constructs, in which SNP intervals were attached to the *KRT1* promoter, and performed transient expression studies. The constructs consisted of the low- and high-expressing *KRT1* promoters alone (numbers 1–2 in Figure 3) and combined with one of nine SNP intervals (containing either the low- or high-expressing alleles): the protein-binding SNPs 5, 11, 17, 23, and 28 (numbers 3–12 in Figure 3) or control SNPs 7, 14, 22, and 27 (numbers 13–20 in Figure 3). The promoter sequences examined were ~ 600 bp in length and the SNP intervals averaged ~ 350 bp in length (Table S4). The control SNP alleles were chosen because they differ between the low- and high-expressing haplotypes but did not bind protein under stringent conditions in the EMSA assay. The construct containing the high expressing version of the *KRT1* promoter alone was used as a control against which the expression levels of all the other constructs were compared. 15% changes in expression compared with the high-expressing *KRT1* promoter alone equal 2 standard deviations and thus are significant (*p* = 0.05).

We first determined whether the activities of the paired low- and high-expressing constructs were different from one another. The activities of the two constructs containing the low- and high-expressing *KRT1* promoters alone were not significantly different from one another (numbers 1–2 in Figure 3). Likewise, the activities of the paired low- and high-expressing constructs containing control SNPs 7, 14, 22, and 27 (numbers 13–20 in Figure 3) were similar to each other. The activities of two of the paired low- and high-expressing constructs containing protein-binding SNPs 5 and 11 (numbers 3–6 in Figure 3) are similar to each other. However, the activities of the other three paired low- and high-expressing constructs containing protein-binding SNPs 17, 23, and 28 (numbers 7–12 in Figure 3) are different from one another by 14%, 18%, and 23%, respectively. These data suggest that the low- and high-expressing intervals containing SNPs 17, 23, and 28 have different affinities for transcriptional regulators.

We then compared the activities of the low- and high-expressing constructs to the activity of the high-expressing *KRT1* promoter alone. The activities of the low- and high-expressing constructs containing control SNPs 7, 22, and 27 were only 2%–8% different from the activity of the *KRT1* promoter alone (numbers 13–14 and 17–20 versus 1 in Figure 3). The activities of the low- and high-expressing constructs containing control SNP14 (numbers 15–16 versus 1 in Figure 3) were both approximately 15% different from the *KRT1* promoter alone. These data suggest that when a random sequence interval of ~ 350 bp is inserted in front of the *KRT1* promoter, the majority of the time the construct will not have significantly increased activity.

The activities of all five pairs of low- and high-expressing constructs containing protein-binding SNPs were significantly different from the *KRT1* promoter alone (numbers 3–12 versus 1 in Figure 3). The constructs containing SNPs 11, 17, and 28 intervals have significantly less activity than the *KRT1* promoter alone. The activities of the low- and high-expressing SNP11 interval constructs were both approximately 30% less than that of the *KRT1* promoter alone. The activity of the low-expressing SNP17 interval construct was 29% less, while the high-expressing construct was 15% less than the *KRT1* promoter alone. The activity of the low-expressing SNP28 interval was 42% less while the high-expressing construct was 19% less than the *KRT1* promoter alone. These data suggest that the intervals containing SNPs 11, 17, and 28 bind transcriptional inhibitors, with the low-expressing SNP17 and SNP28 sequences having a higher affinity for the inhibitors than the corresponding high-expressing intervals.

The constructs containing SNPs 5 and 23 have significantly greater activity than the *KRT1* promoter alone. The activities of the low- and high-expressing SNP5 interval constructs were both approximately 35% greater than that of the *KRT1* promoter alone. The activity of the high-expressing SNP23 interval construct was 38% greater while the low-expressing construct was 20% greater than the *KRT1* promoter alone. These data suggest that the sequences in the SNPs 5 and 23 intervals bind transcriptional activators, with the high-expressing SNP23 sequence having a higher affinity for the activator than the low-expressing SNP23 sequence.

### Interactions of SNP Regulatory Intervals

Next we examined whether combining the SNP17 and SNP28 intervals into a single construct would result in a greater decrease in activity of the *KRT1* promoter than either interval alone. We chose to examine these two SNP intervals because they both bind transcriptional inhibitors, and the activities between the paired low- and high-expressing constructs are significantly different. Two additional constructs were generated, with the low-expressing SNP17 and SNP28 intervals combined with the low-expressing *KRT1* promoter and the high-expressing SNP17, and SNP28 intervals combined with the high-expressing *KRT1* promoter (numbers 21–22 in Figure 3). The combined low-expressing construct had 53% less activity than the *KRT1* promoter alone, which is 24% and 11% less activity than the low-expressing SNP17 and SNP28 constructs (numbers 22, 8, 12 versus 1 in Figure 3, respectively). And the combined high-expressing construct had 24% less activity than the *KRT1* promoter alone, which is 10% and 5% less activity than the high-expressing SNP17 and SNP28 constructs (numbers 21, 7, 11 versus 1 in Figure 3, respectively). Thus, when the SNP17 and SNP28 intervals are combined in a single construct, the *KRT1* promoter activity is repressed to a greater extent than that observed for either interval alone.

Although the two constructs containing either the low- or high-expressing *KRT1* promoters alone were similar in activity, we reasoned that variants in the promoter interval could be interacting with the protein-binding SNP intervals. To examine this possibility, we generated four additional constructs with the low-expressing *KRT1* promoter combined with the high-expressing SNP17 or SNP28 intervals and the high-expressing *KRT1* promoter combined with the low-expressing SNP17 and SNP28 intervals (numbers 23–26 in Figure 3). The high-expressing promoter combined with the low-expressing SNP17 or SNP28 intervals had respectively 3% and 19% greater activity than the low-expressing promoter combined with these same intervals (numbers 23 versus 8, and 25 versus 12 in Figure 3). Whereas the constructs containing the low-expressing promoter combined with the high-expressing SNP17 or SNP28 intervals both had respectively 10% and 18% less activity then the high-expressing promoter combined with the same intervals (numbers 24 versus 7, and 26 versus 11 in Figure 3). These data suggest that the degree of transcriptional repression is dependent on interaction between the repressors binding to the SNP intervals and the *KRT1* promoter itself, with the low-expressing version of the promoter producing a greater inhibitory effect. Thus, polymorphisms in the promoter interval, such as SNP9 and SNP10 (Figure 1), as well as other variants not yet identified, may also play a role in the differential expression of *KRT1* low- and high-expressing haplotypes.

### Decoy Oligonucleotides

To confirm that the inhibitory effects of the ~ 350-bp SNP17 and SNP28 intervals were due to sequences containing the SNPs, we performed double-stranded DNA decoy oligonucleotide assays [28]. When double-stranded DNA decoy oligonucleotides are transfected into cells they compete with regulatory sequences for binding transcription factors and therefore enhance or reduce transcriptional activation. The decoy oligonucleotides, O17_H_, O17_L_, O28_H_, and O28_L_, used to compete with the SNP17 and SNP28 reporter constructs for transcriptional factors, are shown in Figure 3. The activity of the construct containing the high-expressing version of the *KRT1* promoter alone co-transfected with decoy oligonucleotide O17_H_ (number 27 in Figure 3), was used as the control against which the expression levels of SNP17 interval-containing constructs were compared (numbers 28–32 in Figure 3). The activity of this same construct co-transfected with decoy oligonucleotide O28_H_ (number 33 in Figure 3) was used as the control against which the expression levels of SNP28 interval-containing constructs were compared (numbers 34–38 in Figure 3). Co-transfection of SNP17 and SNP28 decoy oligonucleotides with their corresponding constructs (numbers 29–30 and 35–36 in Figure 3) reversed the inhibitory effects of these intervals on the *KRT1* promoter activity (numbers 7–8 and 11–12 in Figure 3). These results imply that the 25-bp regions immediately surrounding SNPs 17 and 28 are responsible for the observed inhibitory effects of the SNP17 and SNP28 intervals on the *KRT1* promoter activity.

Interestingly, the low-expressing SNP17 construct co-transfected with the low-expressing SNP17 allele decoy had 31% greater activity than the control (number 27 versus 30 in Figure 3). When the low-expressing SNP17 allele decoy was co-transfected with the high-expressing construct, and vice versa (numbers 31–32 in Figure 3), the high-expressing construct had 52% greater activity than the control. These data indicate that the low-expressing SNP17 decoy binds the transcriptional repressor more tightly than the high-expressing SNP17 decoy. The observed increased activity of the SNP17 constructs in the presence of the O17_L_ compared with the control is surprising. One explanation for these results is that if the repressor does not bind to the 475-bp SNP17 interval (Table S4), then a binding site for a transcriptional activator becomes available or a DNA conformation change occurs, resulting in an enhancement of the *KRT1* promoter activity. When the low-expressing SNP28 allele decoy was co-transfected with the high-expressing construct, and vice versa (numbers 37–38 in Figure 3), the high-expressing construct had 17% greater activity and the low-expressing construct had 17% decreased activity compared with the control. These data are consistent with the results observed for the SNP17 decoy tests, suggesting that the low-expressing SNP28 decoy binds a repressor more tightly than the high-expressing decoy, and that by preventing the repressor from binding the 414-bp SNP28 interval allows for the binding of an activator or a DNA conformation change, and thus increased construct activity over the control.

### Comparative Sequence Analysis of the 26-Kb *KRT1* Haplotype Block

To further characterize the intervals containing the five *cis*-regulatory SNPs we performed a comparative analysis between human and mouse sequences to determine if they are evolutionarily conserved. We obtained the nucleotide sequence of the 26-kb *KRT1* haplotype and aligned it to the orthologous mouse interval using VISTA browser (http://pipeline.lbl.gov/cgi-bin/gateway2). The alignment of the human and mouse intervals revealed 35 conserved sequences (≥ 100 nucleotides in length and ≥ 70% identity). Of these 35 conserved sequences in the 26-kb haplotype block, 19 overlap exons or UTRs from *KRT1* and *KRT1B,* and 16 are present in non-coding regions. Considering all 29 SNPs in the 26-kb *KRT1* interval, four occur within the 35 conserved sequences (SNPs 2, 12, 18, and 28) (Figure 1). Two of these are present in protein-encoding sequences (SNP2 resides in exon 9 of human *KRT1* and SNP28 resides in exon 2 of human *KRT1B*), and the other two (SNP12 and SNP18) are in conserved non-coding sequences. SNPs 12 and 18 have the same alleles in the *KRT1* low- and high-expressing haplotype patterns, and therefore are not likely to be involved in *KRT1* differential allelic expression. It is interesting to note that the SNP28 interval appears to have dual functions as both a transcriptional regulatory sequence inhibiting *KRT1* promoter activity and an exonic sequence in the *KRT1B* gene [15]. The other four *cis*-regulatory SNPs, SNP5, SNP11, SNP17, and SNP23, are in sequences that are not evolutionarily conserved between human and mouse.

We aligned the *cis*-regulatory SNP intervals and the exonic SNP2 sequence to the chimpanzee genomic intervals to determine which allele (the low- or high-expressing) reflects the ancestral sequence. Four of the *cis*-regulatory SNP intervals containing SNPs 5, 11, 17, and 28 could be aligned unambiguously to chimpanzee sequences, in each case the SNP allele with the lower affinity for the transcription factor was the derived one. Thus, the high-expressing SNP5 allele and the low-expressing SNP11, SNP17, and SNP28 alleles were the ancestral ones. The high-expressing exonic SNP2 was the ancestral sequence. Based on these data, both the low- and high-expressing haplotypes are derived, and haplotype pattern 3 (Table 3) is the ancestral one.

## Discussion

Our aim was to discover and characterize the regulatory sequences responsible for the extreme allele-specific expression differences of *KRT1* in human white blood cells. In all individuals expressing *KRT1* and heterozygous for exonic SNP2, the same allele is always expressed at a significantly higher level then the other allele. These data suggest that the *KRT1* allelic-expression differences likely result primarily from *cis*-regulatory polymorphisms in strong linkage disequilibrium with exonic SNP2. We determined that all nine *KRT1* exons as well as ~22 kb of sequences upstream of the gene are contained within a single haplotype block. The high-expressing *KRT1* exonic SNP2 allele maps to haplotype pattern 2, while the low-expressing SNP2 allele maps to haplotype pattern 1, suggesting that *cis*-regulatory variants differing between these two haplotypes are likely responsible for the majority of the allele-specific expression differences.

Examining SNPs whose alleles differ between the low- and high-expressing *KRT1* haplotypes using a variety of experimental and computational methods, we identified five *cis*-regulatory polymorphisms. SNP5 and SNP23 *cis*-regulatory intervals act as positive regulators of the *KRT1* promoter in luciferase reporter assays, while SNP11, SNP17, and SNP28 *cis*-regulatory intervals act as negative regulators. Consistent with these data is the fact that SNP11 and SNP17 are present in predicted binding sites for ZNF143 and ZEB, respectively; both known to act as negative transcriptional regulators. And SNP23 is present in a predicted binding site for AML-1, a known positive transcriptional regulator. EMSA and chromatin immunoprecipitation assays suggest that ZEB and AML-1 respectively bind the SNP17 and SNP23 intervals in vivo.

Our study shows that the extreme allele-specific expression differences of *KRT1* result from the haplotypic combinations of the five *cis*-regulatory polymorphisms that differ between the low- and high-expressing patterns. The high-expressing alleles of SNP5 and SNP23 bind more protein in the EMSA than the low-expressing allele. In addition, the high-expressing SNP23 allele exhibited an almost 2-fold increase in the *KRT1* promoter activity compared with the low-expressing allele in the luciferase reporter assay. Thus, for both SNP5 and SNP23 the high-expressing alleles appear to have higher affinities for transcriptional activators than the low-expressing alleles. On the other hand, the low-expressing alleles of SNP11 and SNP17 bind more protein in EMSA than the high-expressing allele. In the luciferase reporter assays the low-expressing SNP17 and SNP28 constructs have approximately 2-fold less *KRT1* promoter activity then the high-expressing allele constructs. Thus, our data indicate that for SNP11, SNP17, and SNP28 the low-expressing alleles have higher affinities for transcriptional repressors than the high-expressing alleles. Additionally, when combined, the SNP17 and SNP28 intervals result in a greater reduction of *KRT1* promoter activity then that observed for either interval alone. And interactions between the individual SNP17 and SNP28 intervals with the *KRT1* promoter(s) suggest that there are functional polymorphisms in the promoter region resulting in less activity for the low-expressing version. It is important to note that in addition to these functional promoter variants other *cis*-regulatory polymorphisms may exist in the *KRT1* haplotype block and/or adjacent haplotype blocks that are also involved in the extreme differential *KRT1* allelic expression. However, the haplotypic combinations of the five *cis*-regulatory polymorphisms that we identified and characterized in this study can readily explain a large fraction of the observed allele-specific *KRT1* expression differences.

Previous studies examining allele-specific expression differences have focused on analyzing single SNPs or SNPs grouped in a short interval, such as a promoter [29–34]. Thus, our study provides important new insights into the complexities of the molecular mechanisms underlying allele-expression differences. The fact that each of the five *cis*-regulatory SNPs we characterized contributes to just a fraction of the observed variation indicates that allele-specific expression is itself a complex trait. Interestingly, the finding that allelic-expression differences can result from the interaction of multiple *cis*-regulatory SNPs may explain the difficulties researchers frequently encounter when trying to discover the “causative SNP” underlying a linkage peak or in an interval identified as associated with a trait in a genetic study.

It is generally well proven that non-coding sequences conserved between humans and mice can represent functional regulatory elements [35–37]. However, in a previous study we demonstrated that functional *cis*-regulatory sequences in humans can be missing in other mammals, even closely related primate species [38]. Based on functional data in this study we proposed that this class of *cis*-regulatory sequences represent rapidly evolving elements that are responsible for gene expression differences between species. Since the *cis*-regulatory SNPs identified here are involved in intra-species gene regulatory differences, the fact that four of the intervals, SNP5, SNP11, SNP17, and SNP23, are not evolutionarily conserved between humans and mice is consistent with our previous observations and hypothesis. The fact that the SNP28 interval appears to have dual functions as a *cis*-regulatory sequence and an exonic sequence in the *KRT1B* gene raises the possibility that transcription of the *KRT1* and *KRT1B* genes is linked by a novel mechanism.

*KRT1* has not previously been shown to have a functional role in white blood cells, and hence we are unable to state whether or not the observed expression differences between the low-expressing and high-expressing haplotype patterns have physiological relevance. Interestingly, a recent study indicates that allele-specific expression differences observed in white blood cells can be associated with physiological relevance in other tissues [39]. The investigators of this study identified two genes with allelic-expression differences in white blood cells isolated from osteoarthritis patients and those isolated from control individuals, and they also showed that these same two genes contain 5′ SNPs with statistically significant association with osteoarthritis. *KRT1* is expressed in the basal layer of the epidermis and plays a major role in the differentiation and function of keratinocytes [11]. *KRT1* expression is down-regulated in keratinocytes in response to wounding [40,41]. This down-regulation of *KRT1* expression is thought to be necessary for keratinocytes to make the morphological changes required for migration [40,41] into the wound site. Based on the functional role of *KRT1* it is interesting to hypothesize that the allele-specific expression differences observed in human white blood cells may be associated with keratinocyte migration rates in response to wounding. In theory, if keratinocytes homozygous for the low-expressing haplotype pattern down-regulate *KRT1* expression more quickly than those homozygous for the high-expressing pattern, they should migrate sooner in response to wounding.

## Materials and Methods

### Isolation of DNA and RNA from white blood cell samples.

Thirty-six anonymous individuals were randomly selected at the Stanford Blood Center (Palo Alto, California, United States). White blood cells were isolated from 35–37 ml buffy-coats (white blood cell-enriched blood samples) by centrifugation in Ficoll density medium (Amersham Pharmacia, Little Chalfont, United Kingdom). RNA and DNA were purified using Trizol Reagent (Invitrogen, Carlsbad, California, United States) according to the manufacturer's instructions. Each sample yielded between 200 μg −400 μg of RNA and ~ 1 mg of DNA. The RNA was treated with DNase I, purified again by phenol-chloroform extraction and ethanol precipitated. cDNA was generated by reverse transcription of the RNA using SuperscriptII RT (Invitrogen) in the presence of random hexamers, followed by RNaseH treatment to eliminate the RNA. Both DNA and cDNA were diluted to 20 ng/ μl for use as templates in PCR reactions.

### Differential expression of *KRT1* in white blood cell samples.

We used real-time PCR methods described by Germer et al. [42] to determine whether the *KRT1* alleles were differentially expressed in the white blood cell samples from individuals heterozygous for *KRT1* exonic SNP2. We used two allele-specific forward primers: 5′ GTGGCAGTTCCAGCGTGA 3′ and 5′ GTGGCAGTTCCAGCGTGG 3′ with one reverse primer: 5′ GCATCTGGTTACTCCGGA 3′ in both combinations (one forward primer per reaction) in separate real-time PCR reactions. White blood cell samples with a cycle threshold of greater than 30 were considered to express *KRT1* only at background level.

### Characterization of protein-binding SNP intervals.

We tested the 19 SNPs (excluding exonic SNP2) that differentiate the low- and high-expressing *KRT1* haplotypes for protein binding by performing EMSA. 25-mer oligonucleotides containing the SNPs in the center positions (Table S3) were end-labeled with Biotin-ddUTP by terminal transferase and purified by G-25 spin columns (Amersham). Complementary oligonucleotides were annealed as described previously [28]. Nuclear extracts from a duodenum epithelial cell line (HuTu 80) were isolated using the Nuclear Extraction Kit (Pierce Biotechnology, Rockford, Illinois, United States) according to the manufacturer's instructions. We initially tested a subset of the SNP probes with both stringent (10 mM Tris, 50 mM KCl, 1 mM DTT [pH 7.5], 5 mM MgCl_2_) [43,44] and non-stringent (10 mM Tris, 50 mM KCl, 1 mM DTT [pH 7.5]) binding-buffer conditions. Under non-stringent conditions more than half of the probes tested bound protein, while under stringent conditions we only observed binding to the five reported probes. 2 μl (about 8 μg) nuclear protein extract was incubated with 20 fm biotin-labeled double-stranded oligonucleotides, 1μg poly dI-dC, binding buffer in a 20-μl reaction volume for 20 min at room temperature (Pierce LightShift Chemiluminescent EMSA kit). The reaction mixture was then analyzed by electrophoresis in a non-denaturing 5% acrylamide gel with cold 0.5X TBE running buffer. The DNA-protein complexes in the gel were then transferred to positively charged nylon membrane by electrophoretic transfer in 0.5X TBE at 380 mA for 30–60 min, and detected using the Light-shift Biotin Detection Kit (Pierce Biotechnology). For competition studies, the nuclear extract was pre-incubated with unlabeled double-stranded oligonucleotides (100-fold excess) before adding the biotin-labeled double-stranded oligonucleotides. In the super-shift assays, 2 μg of antibody [anti-ZEB (C-20), anti-ZEB (E-20), anti-AML-1 (N-20), and anti-RXR (ΔN 197)] obtained from Santa Cruz Biotechnology (Santa Cruz, California, United States) were incubated with DNA-protein complexes on ice for 2 h before gel electrophoresis.

### Chromatin immunoprecipitation assay.

Chromatin immunoprecipitation assays were performed using the CHIP Assay Kit according to the manufacturer's protocol (Upstate Biotechnology, Lake Placid, New York, United States) and antibodies obtained from Santa Cruz Biotechnology. Briefly, 1–2 ×10^7^ duodenum epithelial cells (HuTu 80) were fixed with formaldehyde. After cell lysis, the chromatin was sheared with a water-bath sonicator at 30% of maximum power for three 10-s pulses. The cell lysate was then diluted and DNA-protein complexes were immunoprecipitated by the anti-ZEB (C-20), anti-ZEB (E-20), anti-AML-1 (N-20), and anti-RXR (ΔN 197) antibodies. Immunoprecipitated DNA was analyzed for specific enrichment by semi-quantitative PCR using one-third of the eluted material and primer pairs specific for the SNP17 and SNP23 intervals. The primer pairs were as follows: SNP17 forward ACACTGAGCTTGAAGGTCC; SNP17 reverse CTGGAAACAGTGAAAAGGCTG; SNP23 forward CCTGGGAACACAGTGTCTTA; SNP23 reverse GGAGAAACTGAGCTAGGGAA. 26 PCR cycles were used in the analysis to determine if AML-1 binds the interval containing SNP23, and 30 cycles were used in the analysis to determine if ZEB binds the interval containing SNP17.

### Generation of luciferase reporter constructs.

DNA samples known to be homozygous for either the high-expressing *KRT1* haplotype (blood sample number 2) or the low-expressing *KRT1* haplotype (blood sample number 3) were used as PCR templates. Ten sets of PCR primers (Table S4) were used to amplify either the *KRT1* promoter or one of the nine SNP intervals examined, from the two DNA samples. This generated a total of 20 PCR products. The PCR reactions were carried out in 50-μl reaction volumes with 1X PCR buffer, 2mM MgCl2, 0.2 mM dNTP, 20 ng DNA, and 5 units of Taq Gold DNA polymerase. The PCR products were cloned into the TA cloning vector pCR2.1 (Invitrogen). The *KRT1* promoter fragment was digested by *Hind*III from pCR2.1 and ligated into the *Hind*III-site of pGL3-basic vector (Promega, Madison, Wisconsin, United States) to generate the *KRT1* promoter-luciferase reporter constructs, pGL3- PRH and pGL3- PRL, with H and L being the high-expressing and low-expressing alleles respectively. The nine other sets of high- and low-expressing SNP-containing intervals were digested by *Kpn*I and *Xho*I from pCR2.1, gel-purified and ligated into the *Kpn*I and *Xho*I sites of the pGL3-PRH and pGL3- PRL promoter constructs in various combinations to generate SNP region-*KRT1* promoter luciferase reporter constructs pGL3- PH/L SNPnH/L, where *n* is the SNP number.

### Transfection of luciferase reporter constructs.

We transfected each of the 26 luciferase reporter constructs into a duodenum epithelial cell line (HuTu80) that was obtained from ATCC (American Type Culture Collection, Manassas, Virginia, United States) and cultured in MEM alpha medium supplemented with 10% FBS. Although *KRT1* is not known to be expressed in duodenum epithelial cells under normal physiological circumstances, the HuTu80 cells are appropriate to use for examining the effect of the SNP alleles in transcriptional regulation using reporter gene assays because they are of epithelial origin. Approximately 2 × 10^5^ cells/well were seeded in 24-well cell culture plates 24 h before transfection. The cells were simultaneously transfected with one of the 30 pGL_3_-luciferase reporter constructs (0.8 μg) and a pSV- β-galactosidase control plasmid (0.2 μg) (Promega) to use as an internal standard for transfection efficiency using 2-μl Lipofectamine reagent (Invitrogen) according to manufacturer's instructions. For the decoy oligonucleotide analysis 25 pico-moles of double-stranded O17 or O28 decoy oligonucleotides were co-transfected with the reporter constructs [45,46]. After 48 h, the cells were harvested and lysed with reporter lysis buffer. Luciferase and β-galactosidase expression were assayed with the Bright-Glo luciferase assay system (Promega) and the Galactosidase enzyme assay system (Promega), respectively.

### Analysis of luciferase reporter constructs.

The reporter constructs containing only the *KRT1* promoter region were assayed six independent times in triplicate. The other 24 reporter constructs were assayed two or three independent times in triplicate or quadruplicate. Assays were performed on different days, with the promoter-only constructs assayed on each occasion. For each individual transfection the luciferase activity was normalized against the β-galactosidase activity. To allow comparisons between the assays performed on different days, we normalized the results from each day by dividing values for each construct assay by the lowest *KRT1* promoter-only construct value. The high-expressing *KRT1* promoter was the control construct that all other constructs were compared with; its relative luciferase activity was 100% ± 7%, which represents the mean ± standard deviation of 18 independent assays.

### Comparative sequence analysis.

We obtained the nucleotide sequence of the 26-kb haplotype block on human Chromosome 12 (nucleotides 51, 354, 757–51, 381, 206 from NCBI Build 35) from the UCSC Genome Browser Gateway (http://genome.ucsc.edu/cgi-bin/hgGateway). The orthologous mouse interval was identified on Chromosome 15, nucleotides 102, 324, 855–102, 346, 822, by aligning the human Chromosome 12 sequence to mouse genomic sequences (NCBI Build 33) using VISTA browser (http://pipeline.lbl.gov/cgi-bin/gateway2). The alignment of the human and mouse intervals revealed 35 conserved sequences (≥ 100 nucleotides in length and ≥ 70% identity).

## Supporting Information

Table S1 The 29 SNPs in the *KRT1* Haplotype Block (86 KB DOC)

Table S2 *KRT1* Interval Haplotype Patterns in the 23 Haploid Coriell DNA Polymorphism Discovery Resource Samples The *KRT1* haplotype patterns presented in this table were determined as described by Patil et al. [14]. Briefly, we generated 50 ethnically diverse haploid genomes from the Coriell DNA Polymorphism Discovery Resource, using somatic cell radiation. Long-range PCR products approximately 10 kb in length were digested, labeled, and hybridized to a series of SNP discovery arrays. We used a dynamic programming algorithm to partition the SNPs into haplotype blocks. SNPs with minor allele frequencies of at least 10% in the Coriell individuals were included in the map. We required all blocks to satisfy the condition that at least 80% of the haploid samples could be assigned to common haplotype patterns having ≥ 10% frequency. In the genomic region surrounding *KRT1,* 23 ethnically diverse haploid genomes were used for SNP discovery and haplotype map construction.
(102 KB DOC)

Table S3 Forward (F) and Reverse (R) Oligonucleotides Used in the Electrophoretic Mobility Shift Assay (93 KB DOC)

Table S4 Forward (F) and Reverse (R) PCR Primers Used to Generate Luciferase Reporter Constructs. (68 KB DOC)

## **Abbreviations:**

EMSA: electrophoretic mobility shift assay
*KRT1*: keratin-1
SNP: single nucleotide polymorphism
