# Supplementary material for: Allele-Specific KRT1 Expression Is a Complex Trait
Source: PLoS Genet. 2006 Jun 9;2(6):e93. doi: 10.1371/journal.pgen.0020093 (PMC1475705; doi:10.1371/journal.pgen.0020093)
Supplement: Supplementary file 1 [file pgen.0020093.st001.doc]

| **Supplementary Table 1:** The 29 SNPs in the KRT1 haplotype block | | | | |
| --- | --- | --- | --- | --- |
| SNP number | Build 34 position | dbSNP number | Reference allele^1^ | Alternate allele^1^ |
| 1 | 51354757 | rs584843 | T | A |
| 2^2^ | 51355281 | rs14024 | T | C |
| 3 | 51355981 | rs17117940 | C | T |
| 4 | 51356769 | rs2010060 | A | T |
| 5 | 51356955 | rs597685 | G | A |
| 6 | 51357649 | rs2741159 | A | C |
| 7 | 51357827 | rs2741158 | A | G |
| 8 | 51359178 | rs12425650 | A | G |
| 9 | 51360810 | rs2171585 | T | C |
| 10 | 51360860 | rs2171586 | C | T |
| 11 | 51361927 | rs3759191 | T | C |
| 12 | 51362399 | rs3759192 | C | T |
| 13 | 51363592 | rs659010 | G | C |
| 14 | 51364125 | rs711345 | G | A |
| 15 | 51364302 | rs17118001 | C | T |
| 16 | 51365620 | rs1717276 | G | A |
| 17 | 51365641 | rs11170234 | G | A |
| 18 | 51366235 | rs17118028 | G | A |
| 19 | 51366482 | rs1357091 | G | T |
| 20 | 51366599 | rs17118029 | G | A |
| 21 | 51366850 | rs17118035 | C | T |
| 22 | 51366952 | rs7956342 | T | C |
| 23 | 51368520 | rs1567757 | C | A |
| 24 | 51370440 | rs17118044 | T | C |
| 25 | 51373576 | rs7976238 | G | A |
| 26 | 51373716 | rs17118093 | A | C |
| 27 | 51375580 | rs1829637 | G | A |
| 28 | 51377833 | rs1567759 | C | A |
| 29 | 51381206 | rs1877549 | C | T |
| ^1^ The reference and alternate alleles listed here are taken from the RefSeq database and in some cases are the complements of the alleles listed elsewhere in this paper (e.g. Fig. 1)  ^2^ Exonic SNP assayed for allelic expression differences | | | | |
