# Supplementary material for: Allele-Specific KRT1 Expression Is a Complex Trait
Source: PLoS Genet. 2006 Jun 9;2(6):e93. doi: 10.1371/journal.pgen.0020093 (PMC1475705; doi:10.1371/journal.pgen.0020093)
Supplement: Supplementary file 2 [file pgen.0020093.st002.doc]

| Supplementary Table 2: KRT1 interval haplotype patterns in the 23 haploid Coriell DNA Polymorphism Discovery Resource samples | | | | | | | | | | | | | | | | | | | | | | | | | | | | | | |
| --- | --- | --- | --- | --- | --- | --- | --- | --- | --- | --- | --- | --- | --- | --- | --- | --- | --- | --- | --- | --- | --- | --- | --- | --- | --- | --- | --- | --- | --- | --- |
| Haplo-type pattern | Freq.^1^  (%) | SNP number | | | | | | | | | | | | | | | | | | | | | | | | | | | | |
|  |  | **1** | **2**^2,3^ | **3** | **4** | **5**^3^ | **6**^3^ | **7** | **8** | **9** | **10** | **11**^3^ | **12** | **13** | **14** | **15** | **16** | **17**^3^ | **18** | **19** | **20** | **21** | **22** | **23**^3^ | **24** | **25** | **26** | **27** | **28^3^** | **29** |
| 1^4^ | 21.7 | T | G | G | A | T | T | T | A | T | C | T | C | C | A | C | A | G | G | G | G | N | T | C | T | G | A | A | C | T |
| 2^5^ | 30.4 | A | A | G | T | C | G | C | A | C | T | C | C | G | G | C | G | A | G | T | G | C | C | A | T | A | A | G | A | T |
| 3^6^ | 8.7  13.0  4.3  4.3 | A | A | A | T | C | T | T | G | T | C | T | T | G | G | T | G | G | A | G | G | T | T | C | T | G | C | G | C | C |
|  |  | A | A | A | T | C | T | T | G | T | C | T | T | G | G | T | G | G | A | G | G | T | T | C | C | G | C | G | C | C |
|  |  | A | A | G | T | C | T | T | N | T | C | T | C | G | G | C | G | G | G | G | G | N | T | C | T | G | A | G | C | T |
|  |  | A | A | G | T | C | T | T | A | T | C | T | C | G | G | C | G | G | G | G | G | C | T | C | T | G | A | G | C | N |
| 4^7^ | 8.7  4.3 | A | A | G | T | C | T | C | A | C | T | C | C | G | G | C | G | A | G | T | A | C | C | A | T | G | C | G | C | C |
|  |  | A | A | G | T | C | T | C | A | C | T | C | C | G | G | C | G | A | G | T | A | C | C | A | T | N | A | G | N | T |
| 5 | 4.3 | T | A | G | A | T | T | T | N | T | C | N | N | C | A | C | A | G | G | N | G | N | T | C | N | N | A | G | N | N |
| ^1^ Frequency of haplotype pattern in the 23 haploid genomes from the Coriell DNA Polymorphism Discovery Resource (see accompanying Legend)  ^2^ Exonic SNP assayed for allelic expression differences  ^3^ These seven SNPs are KRT1 haplotype block tagging SNPs that together differentiate between the five major haplotype patterns.  ^4^ Pattern number 1 is the low-expressing haplotype pattern as determined by differential allelic-expression of SNP2 in the heterozygous blood samples.  ^5^ Pattern number 2 is the high-expressing haplotype pattern as determined by differential allelic-expression of SNP2 in the heterozygous blood samples.  ^6^ The four haplotypes that make up haplotype pattern number 3 are minor variants on each other.  ^7^ The two haplotypes that make up haplotype pattern number 4 are minor variants on each other. | | | | | | | | | | | | | | | | | | | | | | | | | | | | | | |
