# Supplementary material for: Allele-Specific KRT1 Expression Is a Complex Trait
Source: PLoS Genet. 2006 Jun 9;2(6):e93. doi: 10.1371/journal.pgen.0020093 (PMC1475705; doi:10.1371/journal.pgen.0020093)
Supplement: Supplementary file 3 [file pgen.0020093.st003.doc]

| **Supplementary Table 3:** Forward (F) and reverse (R) oligonucleotides used in the electrophoretic mobility shift assay | |
| --- | --- |
| Name | Sequence^1^ |
| SNP1L-F | TCCAGGTTAGACAGTGCCGTAAACA |
| SNP1L-R | TGTTTACGGCACTGTCTAACCTGGA |
| SNP1H-F | TCCAGGTTAGACAGAGCCGTAAACA |
| SNP1H-R | TGTTTACGGCTCTGTCTAACCTGGA |
| SNP4L-F | GTCAAGGCTCCTACAACCCTTACAG |
| SNP4L-R | CTGTAAGGGTTGTAGGAGCCTTGAC |
| SNP4H-F | GTCAAGGCTCCTTCAACCCTTACAG |
| SNP4H-R | CTGTAAGGGTTGAAGGAGCCTTGAC |
| SNP5L-F | AGCATGATGGCTACATTCTGGAAAC |
| SNP5L-R | GTTTCCAGAATGTAGCCATCATGCT |
| SNP5H-F | AGCATGATGGCTGCATTCTGGAAAC |
| SNP5H-R | GTTTCCAGAATGCAGCCATCATGCT |
| SNP6L-F | CGTTGTGGGTTCAGGCTTAAAAACT |
| SNP6L-R | AGTTTTTAAGCCTGAACCCACAACG |
| SNP6H-F | CGTTGTGGGTTCCGGCTTAAAAACT |
| SNP6H-R | AGTTTTTAAGCCGGAACCCACAACG |
| SNP7L-F | CATGAAGGCACATTCTCTCCAGGGC |
| SNP7L-R | GCCCTGGAGAGAATGTGCCTTCATG |
| SNP7H-F | CATGAAGGCACGTTCTCTCCAGGGC |
| SNP7H-R | GCCCTGGAGAGAACGTGCCTTCATG |
| SNP9L-F | AGCTCACAAGGGCAAGGTGAGAATAGAA |
| SNP9L-R | TTCTATTCTCACCTTGCCCTTGTGAGCT |
| SNP9H-F | AGCTCACAAAGGCAAGGTGAGAATAGAA |
| SNP9H-R | TTCTATTCTCACCTTGCCTTTGTGAGCT |
| SNP10L-F | AAGCACGGAGAATTGCACCTGCTGTGCA |
| SNP10L-R | TGCACAGCAGGTGCAATTCTCCGTGCTT |
| SNP10H-F | AAGCACGGGGAATTGCACCTGCTGTGCA |
| SNP10H-R | TGCACAGCAGGTGCAATTCCCCGTGCTT |
| SNP11L-F | AGAGTACCCAGCATGCATTGCAATG |
| SNP11L-R | CATTGCAATGCATGCTGGGTACTCT |
| SNP11H-F | AGAGTACCCAGCACGCATTGCAATG |
| SNP11H-R | CATTGCAATGCGTGCTGGGTACTCT |
| SNP13L-F | CACTAACATCTACAGGCAGCAACTA |
| SNP13L-R | TAGTTGCTGCCTGTAGATGTTAGTG |
| SNP13H-F | CACTAACATCTACACGCAGCAACTA |
| SNP13H-R | TAGTTGCTGGCTGTAGATGTTAGTG |
| SNP14L-F | CCTTCATCTCTCACTCAAGCAGGCA |
| SNP14L-R | TGCCTGCTTGAGTGAGAGATGAAGG |
| SNP14H-F | CCTTCATCTCTCGCTCAAGCAGGCA |
| SNP14H-R | TGCCTGCTTGAGCGAGAGATGAAGG |
| SNP16L-F | TCACATGGAAGAAGAGATCACAGGT |
| SNP16L-R | ACCTGTGATCTCTTCTTCCATGTGA |
| SNP16H-F | TCACATGGAAGAAGGGATCACAGGT |
| SNP16H-R | ACCTGTGATCCCTTCTTCCATGTGA |
| SNP17L-F | GCCTCAGGTGAGTCCGGTGATGCAC |
| SNP17L-R | GTGCATCACCGGACTCACCTGAGGC |
| SNP17H-F | GCCTCAGGTGAGCCCGGTGATGCAC |
| SNP17H-R | GTGCATCACCGGGCTCACCTGAGGC |
| SNP19L-F | TGGTCCCTAGAAGGACTGCAGACCC |
| SNP19L-R | GGGTCTGCAGTCCTTCTAGGGACCA |
| SNP19H-F | TGGTCCCTAGAAGTACTGCAGACCC |
| SNP19H-R | GGGTCTGCAGTACTTCTAGGGACCA |
| SNP22L-F | GTCAGGGCCACATTTTCTGGGACAA |
| SNP22L-R | TTGTCCCAGAAAATGTGGCCCTGAC |
| SNP22H-F | GTCAGGGCCACACTTTCTGGGACAA |
| SNP22H-R | TTGTCCCAGAAAGTGTGGCCCTGAC |
| SNP23L-F | TGGACTCAGAACTACCACATAGGAT |
| SNP23L-R | ATCCTATGTGGTAGTTCTGAGTCCA |
| SNP23H-F | TGGACTCAGAACTAACACATAGGAT |
| SNP23H-R | ATCCTATGTGTTAGTTCTGAGTCCA |
| SNP25L-F | CTGTGCAGGACCGTGGGACAGAGGT |
| SNP25L-R | ACCTCTGTCCCACGGTCCTGCACAG |
| SNP25H-F | CTGTGCAGGACCATGGGACAGAGGT |
| SNP25H-R | ACCTCTGTCCCATGGTCCTGCACAG |
| SNP27L-F | CATGAACCAGAGACACATTCGCCTC |
| SNP27L-R | GAGGCGAATGTGTCTCTGGTTCATG |
| SNP27H-F | CATGAACCAGGGACACATTCGCCTC |
| SNP27H-R | GAGGCGAATGTGTCCCTGGTTCATG |
| SNP28L-F | TTGGAGAACTACATCGGTGACCTGCGGA |
| SNP28L-R | TCCGCAGGTCACCGATGTAGTTCTCCAA |
| SNP28H-F | TTGGAGAACTACATCTGTGACCTGCGGA |
| SNP28H-R | TCCGCAGGTCACAGATGTAGTTCTCCAA |
| ^1^ The SNP nucleotide is colored red | |
