# Supplementary material for: Allele-Specific KRT1 Expression Is a Complex Trait
Source: PLoS Genet. 2006 Jun 9;2(6):e93. doi: 10.1371/journal.pgen.0020093 (PMC1475705; doi:10.1371/journal.pgen.0020093)
Supplement: Supplementary file 4 [file pgen.0020093.st004.doc]

| **Supplementary Table 4:** Forward (F) and reverse (R) PCR primers used to generate luciferase reporter constructs. | | |
| --- | --- | --- |
| Primer Name | Sequence | Amplicon length |
| SNP9,10-F^1^ | CCATCTTGTTCAATGACAGCT |  |
| SNP9,10-R^1^ | ATAATAAAGCTTAAACACTCCTCTACCCCAAG | 617bp |
| SNP5-F | ATAATAGGTACCCAACCCTTACAGACTTGACAT |  |
| SNP5-R | ATAATACTCGAGTCTCCTTCTACCACTCAGCT | 330bp |
| SNP7-F | ATAATAGGTACCAGGAACAAGGGACATCATGAA |  |
| SNP7-R | ATAATACTCGAGCTATCCGGGGGTGGGACA | 157bp |
| SNP11-F | ATAATAGGTACCCAAGTCATTCTGCCTGCTGT |  |
| SNP11-R | ATAATACTCGAGGCTTCTCCCCTTTGAGTCTT | 399bp |
| SNP14-F | ATAATAGGTACCTCTTCCAAGCCCACTTCTAC |  |
| SNP14-R | ATAATACTCGAGCCAGGAAACACACAAGCACA | 353bp |
| SNP17-F | ATAATAGGTACCACACTGAGCTTGAAGGTCC |  |
| SNP17-R | ATAATACTCGAGCTGGAAACAGTGAAAAGGCTG | 475bp |
| SNP22-F | ATAATAGGTACCTGTCCTGTCATGGAGGCCTG |  |
| SNP22-R | ATAATACTCGAGTGAGTGGGACTCAGGTGAGA | 357bp |
| SNP23-F | ATAATAGGTACCCCTGGGAACACAGTGTCTTA |  |
| SNP23-R | ATAATACTCGAGGGAGAAACTGAGCTAGGGAA | 290bp |
| SNP27-F | ATAATAGGTACCTCACTAAGGTCAGGTTCCAC |  |
| SNP27-R | ATAATACTCGAGCACATCTCACAAGGAGTATGG | 367bp |
| SNP28-F | ATAATAGGTACCATCCTGATTCTCTGAGGTCTG |  |
| SNP28-R | ATAATACTCGAGCTTACACACAAACACTGAAGC | 414bp |
| ^1^ SNP9 and SNP10 are contained in the KRT1 promoter | | |
